# Intravaginal electrical stimulation associated with pelvic floor muscle training for women with stress urinary incontinence: study protocol for a randomized controlled trial with economic evaluation

**DOI:** 10.1186/s13063-021-05781-w

**Published:** 2021-11-20

**Authors:** Bianca Manzan Reis, Jordana Barbosa da Silva, Ana Paula Rodrigues Rocha, Richard Eloin Liebano, Patricia Driusso

**Affiliations:** 1 Women’s Health Research Laboratory, Physical Therapy Post-Graduate Program, Federal University of São Carlos, Rod. Washington Luis, km 235, CEP, São Carlos, SP 13565-905 Brazil; 2 Physioterapeutics Resources Laboratory, Physical Therapy Post-Graduate Program, Federal University of São Carlos, São Carlos, SP Brazil

**Keywords:** Urinary incontinence, Physical therapy specialty, Costs and cost analysis, Women’s health, Pelvic floor, Clinical protocols

## Abstract

**Introduction:**

Pelvic floor muscle training (PFMT) exercises and neuromuscular electrical stimulation (NMES) are described as conservative interventions to prevent or treat female stress urinary incontinence (SUI). However, it has not been described yet the effect of PFMT associated to intravaginal NMES which evaluated the cost-effectiveness and cost-utility of treating.

**Aims:**

To evaluate the effects of intravaginal NMES associated with the PFMT protocol on urinary loss and quality of life in women with SUI and to evaluate the cost-effectiveness and cost-utility and pelvic floor muscle in women with SUI.

**Methods:**

Randomized controlled trial study with economic evaluation. Inclusion criteria are woman (biological), aged ≥ 18 years old and with a report of SUI ≥ once/week. Exclusion criteria are presence of vaginal or urinary infection, virginity, being in the gestational or puerperium period, or neurological disease. Participants will undergo physical therapy assessment and intervention: anamnesis, pelvic floor muscle assessment by vaginal palpation and manometry (Peritron^TM^), questionnaires (Short-Form 6 Dimensions—Brazil (SF-6D), King's Health Questionnaire (KHQ) and King´s Health Questionnaire for Scoring Algorithm), health costs, and voiding diary. Participants will be randomly allocated into 3 groups: CG (control group), IG 1 (intervention group 1, PFMT), and IG2 (intervention group 2, PFMT + NMES). The statistical analysis will be performed by intention to treat, and multivariate analysis of mixed effects will be used to compare outcomes. Effect size measurements will be calculated and will be provided by Cohen’s *d* test. A significance level of 5% will be adopted. Additionally, the incremental cost-effectiveness and incremental cost-utility ratios will be used.

**Discussion:**

This protocol can corroborate with the literature in order to identify the effect of techniques, based on the possibility of confirming the hypothesis that the NMES associated with PFMT performed concurrently will be the best treatment option; considering the effectiveness, cost-effectiveness, and cost-utility analysis, it will be used as an option for optimization of the treatment of SUI.

**Trial registration:**

Brazilian Registry of Clinical Trials (ReBEC) ID: RBR-6gtzg4. Registered on September 3, 2019.

## Introduction

The International Continence Society defines urinary incontinence (UI) as any involuntary loss of urine [1]. UI is defined as stress urinary incontinence (SUI) when the loss of urine is associated to an effort or a physical exertion, cough, or sneeze. The prevalence of SUI between women range from 3 to 25%, and elderly women seem to be the most affected by SUI [2]. Changes in habits, physical inactivity, and social isolation may be associated to SUI, since women may feel embarrassed due to episodes of loss of urine during some activities [3–5]. In addition, the prevalence of SUI may have an important economic impact, since the use of hygienic protectors and routine care or consultations, as well as surgical interventions for the treatment of SUI, is associated with high costs [6].

Pelvic floor muscle training (PFMT) is considered the first-line of prevention and treatment of SUI, with level 1 of evidence and recommendation A [7–9]. Besides PFMT, neuromuscular electrical stimulation (NMES) is considered another available resource to be applied during the SUI treatment and is indicated as a second line of treatment when its use is combined with PFMT [10]. NMES is used as an attempt to recover urinary continence mechanisms by strength gain of pelvic floor muscles (PFM) [11], once the stimulation of the efferent motor fibers of the pudendal nerve causes a direct contraction of PFM or the striated periurethral musculature, enabling the mechanism of closure of the urethral sphincter [12].

However, a systematic review [10] identified that the effect of NMES associated with other therapeutic methods (drugs, PFMT, and vaginal cones) are inconclusive. Although the application of NMES versus PFMT indicated a cure and improvement of SUI reported by women and improved PFM function in the group that underwent treatment with PFMT compared to NMES, no differences were found in the outcomes related to quality of life and quantification of urinary symptoms. In addition, data related to treatment satisfaction, measures of quality of life of general health status, and economic data were not reported.

Data related to the economic analysis in health costs may be considered a relevant tool to be evaluated during SUI treatment. Studies that aim to identify the cost-effectiveness and cost-utility of a specific intervention may be used by governments’ institutions during the decision-making regarding the best evidence-based intervention that may correct the public health problems according their direct finite resources [13]. In Brazil, there are no estimates regarding annual expenses for the treatment of UI. However, it is known that more than $16 billion are spent on UI treatment in the USA annually [14]. It is expected that the economic analysis may affect directly the development of new guidelines related to the clinical practice of health professionals who treat women with SUI.

Therefore, the primary aim of this study is to evaluate the effects of intravaginal NMES associated with the PFMT protocol on urinary loss and quality of life in women with SUI. The secondary aim is to evaluate the cost-effectiveness and cost-utility and pelvic floor muscle in women with SUI.

The hypothesis of the study is that both treatments (NMES associated with PFMT and PFMT) will present good effectiveness; however, considering greater cost-effectiveness, PFMT will be the best option.

## Methods

### Trial design

This is study is a randomized controlled trial with a blind assessor and economic evaluation. Outcome measures will be assessed at baseline (before treatment begins), on the last day of treatment (after the 10th session), and up to 6 months after the treatment is completed (follow-up). From January 2022, participants will be invited and randomized into three groups by a simple randomization using an electronic system “Randomization Main” and a ratio of 1:1:1. The data for the primary outcome will be collected until the end of 2023 and the follow-up data until the first semester of 2024. This project will follow the guidelines of the *Consolidated Standards of Reporting Trials* (CONSORT) and *Standard Protocol items: recommendations for interventional trials* (SPIRIT) [15] items.

### Ethics

The present research was approved by the Ethics and Research Committee of the Federal University of São Carlos. Before the data collection, each participant will receive clarifications about the research objectives, the anonymity of their data, and the freedom to participate and will sign two copies of the informed consent form. This study was registered in the Brazilian Registry of Clinical Trials (ReBEC) under the number http://www.ensaiosclinicos.gov.br/ ID: RBR-6gtzg4, date of registration: September 03, 2019.

After the inclusion of the participant in the study, the subjects will receive a numeric code in order to keep the personal information unidentifiable. In addition, information regarding the telephone number, cell phone, and home address will be kept under the care of the researcher responsible for the project and will not be disclosed in scientific events or papers.

In any case of change, participants will be communicated by the responsible researcher after changes were performed at the Brazilian Registry of Clinical Trials (*Registro Brasileiro de Ensaios Clínicos*—REBEC).

### Participants and settings

The clinical trial will be conducted at the Women’s Health Research Laboratory, Physical Therapy Department, Federal University of São Carlos, Brazil. Participants will be invited through an informal invitation on social media sites (e.g., Facebook and Instagram), leaflets, newspapers, websites, and radios. After selecting the participants, a subjective interview will be conducted in order to assess the sample eligibility.

This study will include women (biological), aged ≥ 18 years, with a report of SUI ≥ once a week [16], with SUI symptoms that will be determined by the affirmative answer to the following *King’s Health Questionnaire* (KHQ) question [17]: “Did you involuntarily lose urine associated with coughing, sneezing, exercise or weight lifting in the last month?” Participants will be excluded if they present vaginal or urinary infection, if they were virgins, and if they were pregnant or in the puerperium period for at least 1 year, are women with neurological disease, and are participants with the incapacity to perform a PFM contraction.

### Measurement of results, primary and secondary outcomes

Outcome measures will be evaluated in several stages: at the beginning (before the beginning of interventions) and at the end of 10 sessions (subjective interview, pelvic floor muscle assessment, KHQ, Short-Form 6 Dimensions- Brazil, King´s Health Questionnaire for Scoring Algorithm and voiding diary). During the follow-up, the outcomes measures will be evaluated by telephone for 6 months. The economic evaluation will be performed during the 6 months of follow; however, at the 3rd and 6th follow-up assessment, we will apply the KHQ, Short-Form 6 Dimensions—Brazil, King’s Health Questionnaire for Scoring Algorithm, additional to the economic evaluation.

The primary outcomes will be urinary symptoms and impact of UI on quality of life. The secondary outcomes will be health cost analysis and PFM function. The impact of UI will be carried out using the KHQ and Short-Form 6 Dimensions—Brazil (SF-6D) questionnaires. Urinary symptoms will be assessed by the voiding diary that will be given to the participant. Cost analysis will be investigated by questionnaires and the PFM function will be assessed by vaginal palpation and manometry.

### Evaluation tools

The objectives of the study will be explained by the responsible researcher, who will clarify all doubts related to the research, and the participants who agree to participate will sign the informed consent form. After the informed consent form was signed, one physiotherapist responsible for the participants’ evaluation will carry out an subjective interview that will include questions related to the personal data, sociodemographic, urogynecology, and obstetric aspects, such as age, profession, years of study, marital status, presence of any disease, urinary complaints, hormone replacement therapy, gestational history, pelvic or abdominal surgery, current urinary tract infection, and previous UI treatment.

Subsequently, the PFM function assessment will be performed by vaginal palpation and manometry. The participant will be ask to position herself in supine position with her hips and knees flexed at 45° and feet supported [18]. To perform the evaluation, the examiner will wear disposable gloves and use lubricant gel. PFM function will be assessed by bidigital vaginal palpation, associating the PERFECT (Table 1) and using the graduation in accordance with the Modified Oxford Scale (MOS) (0 = absence of contraction; 1 = flicker; 2 = weak; 3 = moderate; 4 = good; 5 = strong) [18]. Intra-rater reliability of bidigital vaginal palpation performed by the examiner of the present study was already reported and was considered substantial (κw = 0.75) [19].

**Table 1:** PERFECT scheme

**P**	“Power”: evaluation of a maximal voluntary contraction, graduated by MOS scale
**E**	“Endurance”: time (in seconds) that participant can sustain the contraction with the same degree obtained during the “P” item assessment, considering the maximal time of 10 s
**R**	“Repetitions”: repetition of the “E” item task, performing a 4-s rest between each repetition. The largest number of repetitions is 10
**F**	“Fast contractions”: assessment of the largest number of vigorous and quick contractions (“contracting and relaxing”) until fatigue performed by the participants, with the same strength degree graded in “P” item. Limited to 10 PFM contractions.
**ECT**	“Every Contraction Timed”: the time and sequence of events mentioned above will be timed and recorded

Participants will be instructed to perform the PFM contraction with the following verbal commands: (i) “Contract the pelvic floor muscles as if you are holding the urine”; (ii) “Make a movement with the muscles upwards and inwards”; (iii) “Inhale when your muscles are relaxed and exhale when your muscles are contracting”; and (iv) “Try not to contract the abdomen, glutes or leg muscles while contracting the pelvic floor muscles.” In addition, participants will be instructed to perform the highest contraction strength during three repetitions of maximal voluntary contractions, described by “P” item of PERFECT’s scheme. The highest degree of contraction force will be used in statistical analysis. An interval of 1 min will be adopted between repetitions. The assessor will direct verbal words of encouragement (“hold, hold, hold”) during the contraction.

Five minutes after vaginal palpation, manometry will be performed using the Peritron^TM^ manometer (Cardio Design Pty Ltd, Oakleigh, Victoria, Australia), graded from 0 to 300 cmH_2_O, with a vaginal probe (28 × 55 mm) attached. The manometry will be performed at the same positions and by applying the same verbal instruction as the vaginal palpation. Vaginal probe will be introduced in the participant’s vaginal canal (the tube will be coated with an un-lubricated condom and with a neutral lubricating gel). After calibrating the device, three maximal voluntary contractions of 5 s will be requested and recorded [20]. The validity [21] and intra-rater reliability of the Peritron manometer is already reported and considered good (ICC > 0.90) [22].

The related quality of life and urinary impact will be assessed by the KHQ questionnaire validated to Brazilian Portuguese [17]. This questionnaire consists of 30 questions that assess items related to health perception, impact of urinary incontinence, limitations of daily activities, physical and social limitations, personal relationship, emotions, and sleep/energy, in addition to measures of severity. The score of this questionnaire ranges from 0 to 100, and the higher the score, the worse the quality of life. A change in the baseline of at least 5 points (minimally clinically important difference) in the domains cited above indicates significant change and a clinical improvement in health-related quality of life after treatment [23].

To assess the presence of urinary symptoms, participants will be instructed to complete a 24-h voiding diary. This instrument contains questions related to the urinary habits, such as the information on number of urinations, amount of urine eliminated, and occurrence of urinary loss. In addition, the voiding diary will quantify the use of absorbents, diapers, and linings (if used and the number of changes) [24, 25]. Participants will be encouraged to complete the voiding urinary twice: once at the pre-treatment before the beginning of the intervention and at the end of the treatment (10th session). The results from the voiding diary will be analyzed by urine loss frequency, considering the urinary leakage associated to stress (e.g., coughing, sneezing, physical exercise or household activities).

Health costs related to UI will be assess by two different questionnaires: (i) SF-6D, validated to Brazilian Portuguese by Campolina et al. [26], and (ii) *King´s Health Questionnaire for Scoring Algorithm*, elaborated by Brazier et al. [27]. The SF-6D evaluates items related to functional capacity, global limitation, physical aspects, emotional aspects, social aspects, pain, mental health, and vitality. This questionnaire allows to generate utility indexes (adjusted by years of life) from data of effectiveness used in economic analyzes. The *King´s Health Questionnaire for Scoring Algorithm* is based on specific health measures of UI, which assesses utility based on quality of life with a five-dimensional classification: functional limitation, physical limitation, social limitation, emotions, and sleep. This questionnaire presents a score of validity pointed in 0.62. Both questionnaires have a unique score ranging from 0 to 1 (where 0 represents the worst health status and 1 represents the better health status) [27].

The psychometric properties of the instruments and procedures are present in Table 2.

**Table 2:** Psychometric properties of the instruments and procedures

Examination procedure	Description of procedure	Indicator of musculoskeletal dysfunction	Reported psychometric properties
Modified Oxford Scale (MOS)	Vaginal palpation performed by introduce of two fingers inside participant vaginal canal	Subjective evaluation of PFM function	Intra-rater reliability of MOS (Kappa test) [19] Bidigital palpation 0.75 Inter-rater reliability of MOS (Kappa test) Bidigital palpation 0.60
Manometry	Peritron^TM^ manometer: insertion of a probe into the vaginal canal of participant	Objective evaluation of PFM function	Intra-rater reliability 0.96 [28]
KHQ	Score variating 0 to 100 = the higher the score, the worse the quality of life	Impact of urinary loss	Internal consistency 0.87 [17] Reproducibility: evaluated by Pearson’s coefficient for all domains [29] (listed below): Perception of health 0.36 Impact of incontinence 0.60 Limitations of daily activities 0.75 Physical limitation 0.74 Social limitation personal relationship 0.82 Emotions 0.76 Sleep/energy 0.62 Severity measures 0.71 Minimum relevant clinical difference: 5 points [23] Responsiveness: evaluated by Cronbach’s alpha for all domains [30] (listed below): General health perception − 0.76 Impact of incontinence − 1.94 Role limitations − 1.50 Physical limitations − 1.49 Social limitations − 1.33 Personal relationships − 0.76 Emotions − 1.44 Sleep/energy − 0.86 Severity measures − 2.40
SF-6D	Score between 0 to 100 = the higher the score, the better the health status	Quality of life that constructed data about effectiveness, used in economic analyzes	Internal consistency: – Reproducibility intra-rater: evaluated by Pearson’s correlation coefficient for all domains [31] (listed below): Functional capacity 0.8 Physical aspects 0.63 Social aspects 0.75 Emotional aspects 0.44 Pain 0.54 Vitality 0.65 Mental health 0.69 General health status 0.84 Specificity: – Minimum relevant clinical difference: – Responsiveness: 0.404 (performed in women with postoperative pelvic organ prolapse; the value of the total score was used to identify the result of responsiveness through the ANOVA test) [32]
King’s Health Questionnaire for Scoring Algorithm	Score: 0 = worst health status 1 = better health status	Health measures of UI	Internal consistency: – Reproducibility: – Specificity: – Minimizes relevant click difference: – Responsiveness: evaluated by standardized response mean (SRM) for all domains (listed below). The SRM is a version of the effect size that divides the mean change by the standard deviation of the change [27]: Role 0.56 Physical 0.51 Social 0.39 Personal 0.33 Emotions 0.37 Sleep 0.41 Severity 0.50 Valid: –

MOS, Modified Oxford Scale; PFM, pelvic floor muscle; KHQ, King’s Health Questionnaire; SF-6D, Short-Form 6 Dimensions—Brazil; UI, urinary incontinence

A semistructured questionnaire will be used to assess the economic data related to the health services use. In addition, the use of medications, history of medical consultation and/or emergency care, history of hospitalizations related to urinary problems, and surgical procedures will be verified and will be followed up for 6 months. The cost of which treatment for each participant will be assessed according to the use of health services and calculated by the price list of the Brazil’s Unified Health System and according to the physiotherapist’s fees established by the Federal Council of Physiotherapy and Occupational Therapy and also by average of three budgets for private treatment. Costs will be computed with the use of sanitary napkins, diapers and liners, medications, exams related to urinary problems, consultation with health professionals (such as nurses, doctors and/or physiotherapists), and other interventions and surgical procedures.

In order to compose the economic evaluation, the expenses related to materials used during physical evaluations and intervention (such as gloves, condoms, lubricants, disposable sheets, pressure gauge, NMES devices and probes, and the physiotherapist assessment price) will be computed. In addition, to compose the health costs of tertiary care, information related to hospitalization history, surgery history, average hospitalization time, and absenteeism for health reasons will be verified. To convert the procedures into current currency, the values informed by the Brazil’s Unified Health System price tables and/or based on the market average price will be used. Monetary data will be made in the national currency (real) and converted into international currency with an updated conversion rate.

To compose the indirect costs, the participants will be asked about the days of absence from work (paid or unpaid), which will be computed by means of specific price weights for the female gender and transportation to get to the place where the treatment will be carried out. In addition, the distance between the participants home to the location where the treatment will be applied will be computed. Therefore, the price of the means of transport for those using public transport or weighting of the price of the fuel of private transport will be assessed.

### Sample size calculation

The sample calculation was performed to detect an intergroup difference of 5 points at KHQ domains [23] (incontinence impact, daily activities limitations, physical and social limitation, personal relationship, emotions and sleep/energy). An estimated standard deviation of 10 points was adopted, considering three groups, significance level of 5%, power of 85%, and effect size of 0.25. Considering a possible sample loss of 10%, 4 participants will be added per group. Thus, the sample will be composed of 132 women (44 per group).

### Randomization and allocation

**Fig. 1:**
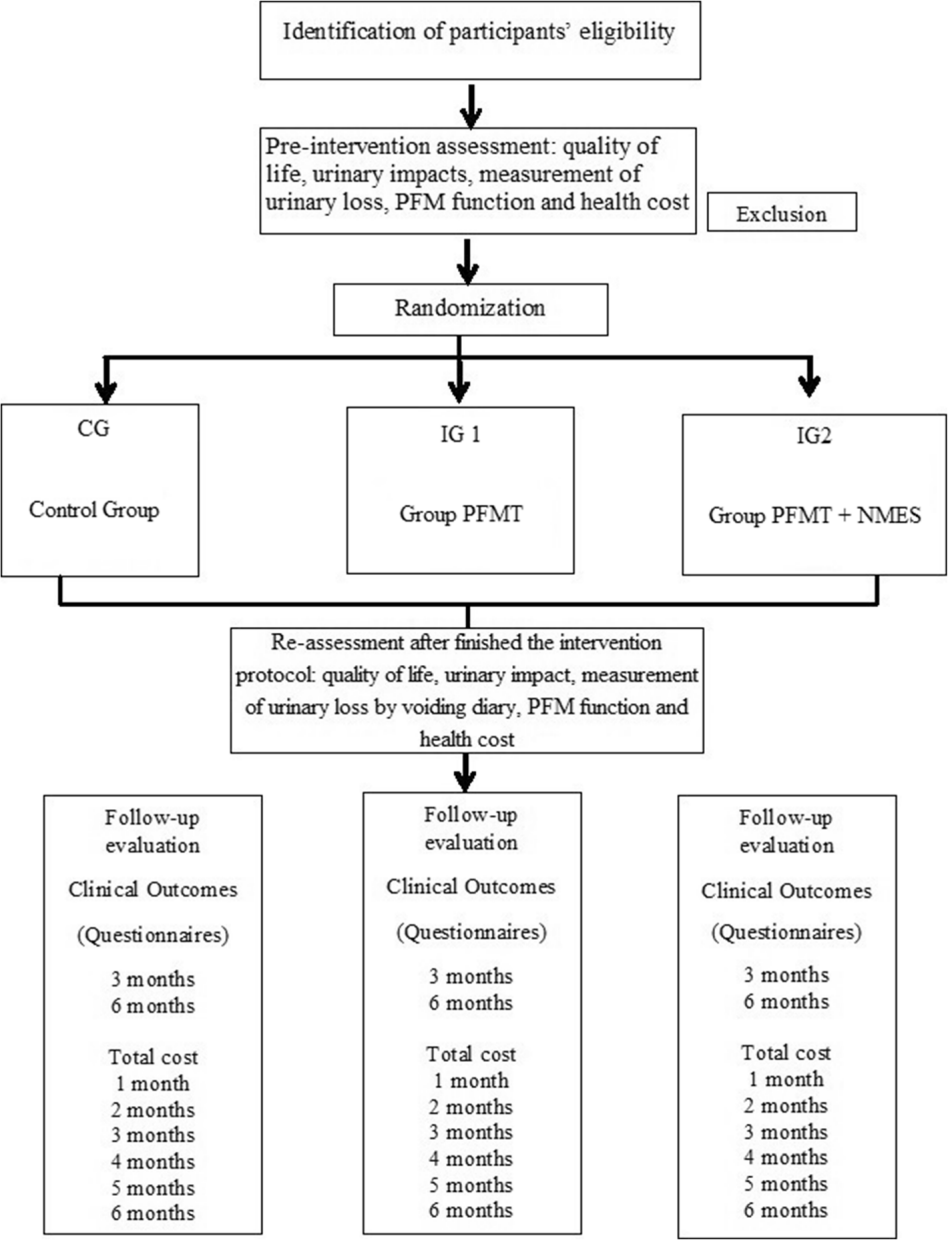
Flowchart of randomized clinical trial. IG1, intervention group 1; IG2, intervention group 2; PFM, pelvic floor muscles; PFMT, pelvic floor muscle training; NMES, neuromuscular electrical stimulation

Once included, the initial assessments will be carried out before the participants are randomized (1:1:1) into three groups (CG (control group without physiotherapist-guidance booklet related to PFMT), IG 1 (intervention group 1, that will perform PFMT with physiotherapist supervision once a week), and IG2 (intervention group 2, that will perform PFMT + NMES with physiotherapist supervision once a week)), using the electronic system “Randomization Main.” One researcher, who will not be involved into the participants’ recruitment or treatment, will perform the randomization by the electronic system and will maintain the randomization numeric sequence inside opaque envelopes (Fig. 1). After the assessment of the eligibility, one physiotherapist responsible for the application of the interventions will open one sealed opaque envelope (containing the proposed treatment and the code of “Randomization Main”) and will schedule the date to the beginning of treatment as specified by one of the three groups.

**Fig. 2:**
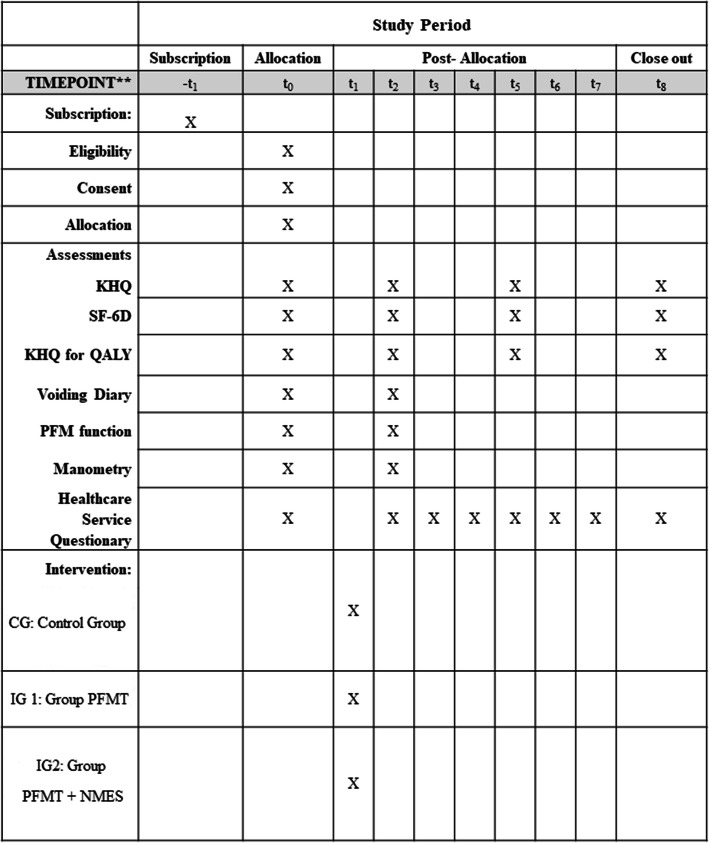
Schedule of registrations, interventions, and evaluations. IG1, intervention group 1; IG2, intervention group 2; PFMT, pelvic floor muscle training; NMES, neuromuscular electrical stimulation; -*t*_1_, subscription of participants; *t*_0_, allocation and initial assessment of participants; *t*_1_, intervention; *t*_2_, re-assessment after finished the intervention protocol; *t*_3_-*t*_8_, follow-up. Asterisks symbol indicates the following: recommended content and elaboration by SPIRIT 2013 for protocols [15]

Two physiotherapists specialist in Women's Health will carry out, guide, and apply the interventions. They will not be involved in the participants’ randomizations, evaluations, or reevaluations. The flowchart is presented in Fig. 1, and the schedule of registrations, interventions, and evaluations are presented in Fig. 2.

### Interventions

Ten treatment sessions will be held, once a week, with a total of 10 weeks of attendance. The participants will be instructed to perform the protocol daily at home. The CG (control group) will receive an orientation booklet for the proposed protocol. The IG1 will perform PFM protocol with physiotherapist supervision 1 time a week and will be instructed to perform the exercise at home without supervision, and the IG2 will perform the PFM protocol (only in supine/lying position) associated with the intravaginal NMES technique with a physiotherapist supervision for 20 min, once a week.

PFM will be performed as the protocol proposed by Bø, Talseth e Holme [33]:

- Positions: lying, kneeling, sitting, and standing;
- Series: 1 series of exercise for each position;
- Repetitions: 12 sustained contractions with 6 s of duration;
- Rest: 6 s;
- After repetitions of sustained contractions, 4 fast contractions are added;
- Intensity: maximal voluntary contraction;
- Session duration: 45 min.

The intravaginal NMES technique, applied to IG2, will be performed with the Dualpex 961 equipment (Quark Medical Products), following the description of the parameters suggested by the study conducted by Barbosa et al. [34]. The participants will remain in supine position, with flexion of the hips and knees. The physiotherapist responsible for the interventions will wear procedure gloves during the entire session and will apply lubricating gel on of the vaginal probe that will be introduced into the participant’s vaginal canal. During the period of NMES application, the physiotherapist will fix the probe manually. At the end of study, vaginal probes will be discarded. The parameters used during NMES application are described in Table 3. In the first 5 treatment sessions, the ON time stimulus will be fixed in 5 s, and the OFF time stimulus will be 15 s. For the last five treatment sessions, the OFF time stimulus will be decreased to 10 s.

**Table 3:** Randomized controlled blinded ES parameters

	1–5th session	6–10th session
Phase duration (μs)	700	700
Frequency (Hz)	50	50
Ramp-up (s)	1	1
Stimulus on time (s)	5	5
Ramp-down (s)	0	0
Stimulus off time (s)	15	10
Duty-cycle ratio	1:3	1:2
Time (min)	20	20

μs, microseconds; Hz, Hertz; s, seconds

During the first five treatment sessions, IG2 will perform 4 series of 12 sustained voluntary contractions of 6 s with 4 fast contractions added to the end of the protocol and 50 s of rest at the end of each series, according the 1:3 duty-cycle ratio. At the last five treatment sessions, participants will perform the same protocol; however, the duty-cycle ratio will decreased to 1:2, and subjects will perform 4 series of 12 sustained contractions for 6 s with 4 fast contractions, with 1 min and 50 s of rest at the end of each series.

**Fig. 3:**
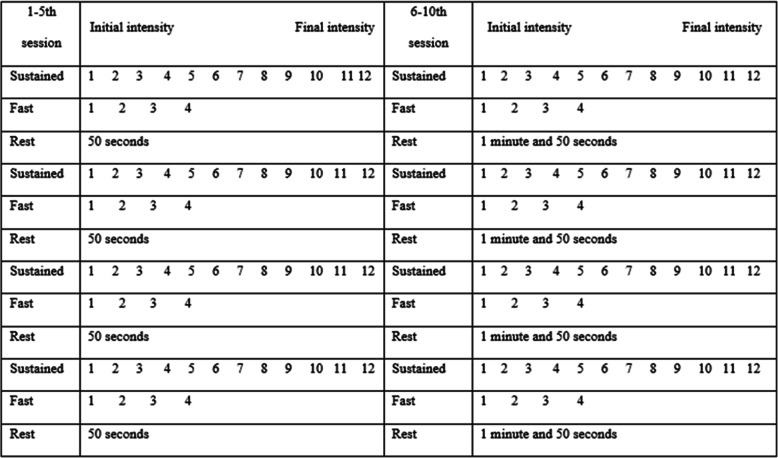
Scheme of the numbers of sustained, fast contractions, and rest time of the sessions of intravaginal electrical stimulation

To perform the contractions protocol, at the beginning of the “on time” (indicating the current flow), the physiotherapists responsible for the interventions will give the following verbal instruction to the participant: “contract, 1, 2, 3, 4, 5, 6, relax.” At the end of each series of sustained contraction, the participants will be instructed to perform fast contractions with the following command “contract-relax, contract-relax, contract-relax, contract-relax.” Four series will be completed as equivalent to the 4 positions proposed by Bø, Talseth e Holme [33] during the PFMT protocol (standing, sitting, kneeling, and lying). During each rest period, participants will be asked if the intensity of NMES can be increased and in all sessions the values of the intensities at the beginning and at the end of the sessions will be recorded. Figure 3 presents the sequence of intervention sessions.

The presence of any adverse effects or pain and discomfort related to the application of the NMES will be considered as the criterion to discontinue the treatment. Participants will be instructed to immediately communicate the researcher responsible for the data collection in any case of adverse effect.

### Blinding

In view of the impossibility of blinding the participants in relation to the interventions, the blinding will happen among the physiotherapists, taking into account the evaluation and intervention. The physiotherapist who will carry out the evaluation will not have the information of the participants’ allocation, and the physiotherapists responsible for guide the interventions will not have the information referring to the evaluations and reevaluations of the participants.

### Minimizing missing data

To avoid and minimize missing data, all participants will receive reminders with the date and time for each session. Physiotherapists responsible for the interventions will also make calls to reinforce the scheduling of sessions. Regarding the evaluations and reevaluations, the blind assessor will contact the participant by telephone, at the end of the 10 treatment sessions, in order to define the best time and date for the reevaluation to be carried out.

### Data management

The data will be collected on paper and stored in folders in an office allocated at the Women’s Health Research Laboratory (LAMU), in Federal University of São Carlos. The data will be entered into the Excel software by the physiotherapist responsible for evaluations, reevaluations, and follow-up. In addition, all the data will be available in a public research bench.

The database and electronic analyzes will be stored on a secure computer server, with personal login access authorized by the principal investigator of the present study. The principal investigator will have access to the complete data set (without the allocation groups), and the co-investigators will have access to data when necessary. Upon completion of the study, all study data and documents will be archived by the main researcher of this study for 5 years at the Physiotherapy Department at Federal University of São Carlos.

### Data monitoring

We will conduct the study without a Data Monitoring Committee (CMD). PFMT, NMES, and home treatments are widely used in clinical practice and have proven to be acceptable. Since there is no CDM, we will not conduct an intermediate analysis during the clinical trial.

### Harms auditing

All participants’ medical records will be carefully evaluated, and all damages and complications related to treatments will be reported, if any. The damage will be classified as adverse events and their severity.

### Auditing

The coordinator of the laboratory where the research will be conducted will not participate in any of the research data collection steps. Therefore, she will be responsible for auditing and access to the final dataset all procedures that will be performed.

### Statistical analysis plan

The statistical analysis will be performed by a researcher without involvement in the evaluations and treatment intervention. The software SPSS 16.0 and R 3.5.1 will be used for the analysis of data. The level of significance will be set at 5%. The normality of the residues will be verified by the Shapiro-Wilk test.

The comparison of SUI, voiding habits, and quality of life between groups CG, IG 1, and IG 2 and between stages (pre-treatment, re-assessment after finished the intervention protocol and follow-up), as well as the interactions between groups, will be performed by means of analysis of variance (ANOVA) of mixed models considering group and time factors for repeated measures, if the residuals present normality with post hoc contrasts (group IG1 and IG2 versus group CG, and group IG1 versus group IG2).

The first statistical procedure will verify, in general and at each evaluation stage (pre-treatment, re-assessment after finished the intervention protocol and follow-up), if SUI urine loss per day related to stress (e.g., coughing, sneezing, physical exercise or household activities) and quality of life differ into the three groups. The null hypothesis is that all groups will present equal results during outcomes assessments. The alternative hypothesis is that IG 1 will differ from CG and that IG2 will differ from the CG as well.

The second statistical procedure aims to verify if there is alteration in the SUI, urine loss per day related to stress (e.g., coughing, sneezing, physical exercise or household activities), and quality of life throughout the four stages of study evaluation (pre-treatment, re-assessment after finished the intervention protocol and, follow-up of the 3rd and 6th month) within each of the groups. The null hypothesis is that the behavior is the same throughout the stages in each group. The alternative hypothesis is that there is a difference in the stages in each group.

The quantitative variables will be presented as minimum value, maximum value, mean, median, and standard deviation. Categorical variables will be presented as frequencies and percentages.

Details of the statistical analyzes are as follows:

1. At the interview and physical assessment, the subjective interview record will be applied, which generates qualitative variables that will be presented as frequencies and percentages and quantitative variables that will be presented as minimum value, maximum value, mean, median, and standard deviation. One researcher will perform the vaginal palpation and the manometry assessment, as well as applied SF6-D, KHQ, King´s Health Questionnaire for Scoring Algorithm, health cost analysis form, and 24 h-voiding diary, which will result in quantitative scales that will be presented as minimum value, maximum value, mean, median, and standard deviation.
2. At the end of treatment sessions, vaginal palpation and manometry will be repeated, as well as SF6-D, KHQ, King’s Health Questionnaire for Scoring Algorithm, health cost analysis form, and the 24 h-voiding diary, which will result in quantitative scales that will be presented as minimum value, maximum value, mean, median, and standard deviation.
3. At the 1st to the 6th month of follow-up, the health cost analysis form will be applied, and at the 3rd and 6th month of follow-up, the SF6-D, KHQ, King´s Health Questionnaire for Scoring Algorithm, and health cost analysis form will be completed, which will result in quantitative scales that will be presented as minimum value, maximum value, mean, median, and standard deviation.
4. At the end of the treatment, the ANOVA for repeated measurements will be applied to verify the effects of the three groups and the two stages (pre-treatment and re-assessment after finished the intervention protocol) and the interactions between them according the results related to the vaginal palpation, manometry, SF6-D, KHQ, King´s Health Questionnaire for Scoring Algorithm, health cost analysis form, and 24 h-voiding diary, if the residues present normality. If the residues do not present normality, a non-parametric test will be applied.
5. At the end of the follow-up, the repeated measures ANOVA will be applied to verify the effects of the three groups and the eight stages (pre-treatment, re-assessment after finished the intervention protocol and 1, 2, 3, 4, 5, and 6 months of the follow-up) and the interactions between them considering the SF6-D, KHQ, King’s Health Questionnaire for Scoring Algorithm, health cost analysis form, and 24 h-VD, if the residues present normality. If the residues do not present normality, a non-parametric test will be applied.
6. The cost-utility assessment will be performed by the quality-adjusted life years (QALY), which combines the length and quality of life into a single index number between 0 and 1, where 0 corresponds to a health state judged to be equivalent to death and 1 corresponds to optimal health [35, 36]. To conduct the statistical analysis, the mean between-group cost differences will be corrected for their baseline, and the total costs will be divided by the costs differences for incremental analysis of cost-effectiveness and cost-utility ratios. Subsequently, a deterministic cost-utility value and a probabilistic one will be determined by applying a bootstrapping technique of 5000 interactions and the bias-corrected, in order to compare health costs between groups. The results will be presented with the confidence interval of 95% (95% CI). The likelihood of the cost-effective intervention will be estimated by the cost-effectiveness acceptability curves to compared values other than willingness to pay [37]. The Kaplan-Meier method and the Log-Rank between-group comparison test will be applied in order to assess the sensitivity of the uncertainty of each parameter by assessing the changes in the results in the range of parameter values [38].

## Discussion

This study intends to demonstrate whether if SUI and urinary impact in quality of life decrease after a protocol treatment with home exercise guidance without physiotherapeutic supervision and after a protocol of PFMT alone or associated with intravaginal NMES supervised by one physiotherapist. In addition, it is expected that the present study demonstrate the effectiveness, cost-effectiveness, and cost-utility analysis of each proposed treatment. It will be seen in possible changes related to the SUI symptoms of after 10th treatment sessions and during a 6-month follow-up after the end of the treatment.

PFMT is a considered a technique with level of evidence A for the treatment of SUI. However, studies related to the NMES application during SUI are still inconclusive; however, it presents promising results in the treatment of SUI [10]. All the techniques mentioned in the present study are characterized as simple and with minimal occurrence of adverse effects, which facilitates the acceptance of therapy by women who need assistance. As the three groups included in the present study will be instructed to outperform a PFMT protocol daily at home, the addition of the group that will receive guidance only through the booklet will allow us to assess the true effectiveness of the association of PFMT and intravaginal NMES with professional physiotherapist supervision.

In addition, economic analyzes have been decisive in treatment options and may involve direct and indirect costs, with cost measures in monetary value. Then, we will conduct a study that will allow the identification of clinically important outcomes such as the general effectiveness or cost-effectiveness of NMES for SUI treatment in Brazilian women [39], items that are still not described in the previous literature [10].

According to a recently systematic review about scientific evidence of intravaginal NMES in SUI [10], one of the main findings was the low methodological quality of the studies, which may interfere with its reliability and application in clinical practice. Therefore, the present protocol can corroborate with the literature on the effects investigation of the intravaginal NMES associated with PFMT on SUI in addition to the identification of socioeconomic data.

After the conclusion of this research, the results will be presented in journals through peer-reviewed publications and scientific events, including the International Congress.

## Trial status

The protocol registration was approved on 3 September 2019 (it was updated and approved on February 24, 2020) and is at version 2. The initial recruitment date will be in January 2022, and the approximate date of completion of until the first semester of 2024.

## Data Availability

Not applicable.

## Abbreviations

UI: Urinary incontinence
UUI: Urgency urinary incontinence
SUI: Stress urinary incontinence
MUI: Mixed urinary incontinence
PFM: Pelvic floor muscles
PFMT: Pelvic floor muscle training
ICT: Informed consent term
LAMU: Laboratory of Research in Women’s Health
UFSCar: Federal University of São Carlos
ANOVA: Analysis of variance
KHQ: King’s Health Questionnaire
24 h-VD: 24 h-voiding diary
DMC: Data Monitoring Committee
SF-6D: *Short-Form 6 Dimensions*—Brazil
